# Reactome pathway analysis: a high-performance in-memory approach

**DOI:** 10.1186/s12859-017-1559-2

**Published:** 2017-03-02

**Authors:** Antonio Fabregat, Konstantinos Sidiropoulos, Guilherme Viteri, Oscar Forner, Pablo Marin-Garcia, Vicente Arnau, Peter D’Eustachio, Lincoln Stein, Henning Hermjakob

**Affiliations:** 1 European Molecular Biology Laboratory, European Bioinformatics Institute (EMBL-EBI), Wellcome Genome Campus, Hinxton, UK; 2 Open Targets, Wellcome Genome Campus, Hinxton, UK; 3 Fundación Investigación INCLIVA, Universitat de València, Valencia, Spain; 4 Instituto de Medicina Genomica, Valencia, Spain; 5 Escuela Técnica Superior de Ingenierías, Universitat de València, Valencia, Spain; 6 Institute for Integrative Systems Biology (I2SysBio), Universitat de València-CSIC, Paterna, Valencia Spain; 7 NYU Langone Medical Center, New York, USA; 8 Ontario Institute for Cancer Research, Toronto, Canada; 9 Department of Molecular Genetics, University of Toronto, Toronto, Canada; 10 State Key Laboratory of Proteomics, Beijing Proteome Research Center, Beijing Institute of Radiation Medicine; National Center for Protein Sciences, 102206, Beijing, China

**Keywords:** Pathway analysis, Over-representation analysis, Data structures

## Abstract

**Background:**

Reactome aims to provide bioinformatics tools for visualisation, interpretation and analysis of pathway knowledge to support basic research, genome analysis, modelling, systems biology and education. Pathway analysis methods have a broad range of applications in physiological and biomedical research; one of the main problems, from the analysis methods performance point of view, is the constantly increasing size of the data samples.

**Results:**

Here, we present a new high-performance in-memory implementation of the well-established over-representation analysis method. To achieve the target, the over-representation analysis method is divided in four different steps and, for each of them, specific data structures are used to improve performance and minimise the memory footprint. The first step, finding out whether an identifier in the user’s sample corresponds to an entity in Reactome, is addressed using a radix tree as a lookup table. The second step, modelling the proteins, chemicals, their orthologous in other species and their composition in complexes and sets, is addressed with a graph. The third and fourth steps, that aggregate the results and calculate the statistics, are solved with a double-linked tree.

**Conclusion:**

Through the use of highly optimised, in-memory data structures and algorithms, Reactome has achieved a stable, high performance pathway analysis service, enabling the analysis of genome-wide datasets within seconds, allowing interactive exploration and analysis of high throughput data. The proposed pathway analysis approach is available in the Reactome production web site either via the AnalysisService for programmatic access or the user submission interface integrated into the PathwayBrowser. Reactome is an open data and open source project and all of its source code, including the one described here, is available in the AnalysisTools repository in the Reactome GitHub (https://github.com/reactome/).

## Background

Reactome (http://reactome.org) is a free, open-source, curated and peer-reviewed knowledge-base of biomolecular pathways. It aims to provide bioinformatics tools for visualisation, interpretation and analysis of pathway knowledge to support basic research, genome analysis, modelling, systems biology and education.

Nowadays, pathway analysis methods have a broad range of applications in physiological and biomedical research. On the one hand, based on a given dataset, these methods help researchers to discover which areas of biology, and biomolecules, are crucial to understand the phenomena under study. On the other hand, pathway analysis methods should never be taken as black boxes from where experimental data goes in, and true statements come out, but perhaps more as metal detectors in haystacks helping researchers to find biologically meaningful needles [1].

Pathway analysis methods are mainly used to analyse Omics data obtained from high-throughput technologies. Since the size of the data samples is constantly increasing [2, 3], Reactome offers a set of pathway analysis tools which aim to deal with this scenario and yet provide reliable and accurate results with interactive (seconds) response time for genome-wide datasets.

Here, we are discussing the high performance Reactome implementation of the well established over-representation analysis (ORA) method [4], focussing on the computer science aspect, elaborating on the different data structures and design patterns used to optimise the execution time and reduce the server load.

Initially the focus is on the strengths and weaknesses of keeping the data directly in a relational database and its usage to perform in-database analyses. Then we continue with a detailed explanation of the new pathway analysis approach, and conclude with the presentation of the results and the discussion.

### The relational databases approach

Relational databases are widely used in pathway knowledge-bases for data management; either during curation, the release process or in the final production phase. It is also very common to store the information in third normal form due to its convenience for data integrity assurance [5–7].

Relational databases in their third normal form can be efficient in computational terms. For the above mentioned use cases, however, this approach greatly slows the execution of analysis algorithms, due to the size of the temporary tables for the queries and later projections. For this reason database-based analysis approaches use denormalised versions of the databases instead [8]. The denormalisation process replicates a lot of data to speed up the queries but it may penalise analysis execution time as the original database content grows bigger.

Focusing on the computational side of the problem, the query containment problem is undecidable for relational algebra and SQL, but is decidable and NP-complete for conjunctive queries. In fact, the query containment problem for conjunctive queries is exactly the same problem as the query evaluation problem [9]. When queries tend to be small, NP-completeness is usually considered acceptable but its performance falls when queries tend to be big. In addition, it is also worth considering that creating intermediate tables in memory after executing a “join” statement is one of the heaviest operations for a database engine.

Reactome’s previous implementation of the pathway analysis was based on a denormalised version of the Reactome relational database. Among its limitations were that it provided results only of the higher-level pathways in Reactome, and the lack of programmatic access. In addition, the previous implementation suffered from poor performance mainly due to the fact that, on every analysis request, it connected to the relational database, rather than querying an intermediate in-memory data structure. Thus, the response time of the previous Reactome analysis could reach 5 min, as soon as the user sample included a few hundreds of gene identifiers, causing a high server load that, combined with a number of concurrent analysis requests, affected the stability of the Reactome website and often resulted in outages.

In resources like Reactome, analyses use not only curated data but also extra information and cross-references to other resources that are included in the final version of the database, for example to allow usage of identifiers from other resources than the main ones used by the curators to identify proteins, genes, microRNAs or chemicals. Each major resource uses its own conventions when assigning identifiers, so the problem of mapping the various, potentially unstable, identifiers that refer to identical entities, commonly known as identifiers mapping, constitutes a major challenge. There is a number of resources that aim to provide a solution to this problem, most notably, the Protein Identifier Cross-Reference (PICR) [10], BridgeDB [11] and UniProt [12]. However, Reactome addresses this problem during each release process by cross-referencing every curated entity to other resources. In particular, based on the UniProt or ChEBI identifiers of the curated entities, filled in during curation, Reactome queries Orphanet, Protein Ontology (PRO), IntAct, RHEA, DOCKBlaster, FlyBase, The Human Metabolome Database (HMDB), Zinc, KEGG, UniProt, ENSEMBL, BRENDA and IntEnz to get their cross-references for entities annotated in Reactome. Both, curated and cross-referenced identifiers are included in the analysis lookup table, as explained in the Implementation section.

As the amount of curated data in Reactome grows and the number of cross-references increases due to the inclusion of new resources, the database-based approach does not scale well, so there is a need to implement a new approach to provide fast, accurate and reliable analysis tools to the final users. This new approach is based on the concatenation of different steps, each one resolved via the appropriate data structure, as explained in the next section.

## Implementation

Identifying a convenient data structure to solve a given problem is one of the main factors to achieve a high performance final product. As Skiena explains in [13], picking the wrong data structure for the job can be disastrous in terms of performance but identifying the very best data structure is usually not as critical, because there can be several choices that perform similarly.

Based on the divide and conquer rule, the first step is breaking down the analysis problem into different sub-problems simple enough to be solved in polynomial time by identifying a convenient data structure. Here, the analysis algorithm can be split into four parts: (1) checking whether the user’s protein/chemical identifiers are present in Reactome, (2) for the present ones, finding whether these are parts of complexes and/or sets as well as the species projection, (3) aggregating the found identifiers in the pathways (and super-pathways) where these are present and finally (4) performing the statistical testing to calculate the likelihood that the association between the sample identifiers and the found pathway is due to random chance.

Further on in this section each part is discussed in detail to determine its peculiarities; to expose the chosen data structure and the mechanisms adopted for its improvement; and to show how to connect each step to the following one to come up with the final improved analysis algorithm. Another point of emphasis for optimisation will be the memory usage of each step, so that the filled data structures can be kept in memory to improve the performance of the data traversing algorithms implemented on top of them.

### User sample identifiers search in Reactome

Annotated physical entities (PE) in Reactome can be either single entities or complexes. Single entities include proteins, small molecules, RNA, DNA, carbohydrates, or lipids, whilst complexes consist of a combination of any of the single entities, or polymers synthesized from the single entities. However, apart from these two main categories, curators in Reactome can group related entities into sets. PEs are the building blocks that later on will be used as inputs, outputs, catalysts or regulators in reactions.

Identifiers or accession numbers are used to unequivocally refer to a single entity, but PEs have different slots to hold the main identifier, secondary identifier, cross-references, synonyms and other identifiers. The main identifier slot is always manually annotated by the experts who curate data in Reactome (curators), and the other slots can either be manually filled during curation or automatically populated during the release process. This strategy allows storing identifiers for a wide range of resources: UniProt, ChEBI, Ensembl, miRBase, GenBank/EMBL/DDBJ, RefPep, RefSeq, EntrezGene, OMIM, InterPro, Affymetrix, Agilent, KEGG Compound, Illumina, etc.

Therefore, in the first part of the analysis, the main requirement is to improve the process of finding out whether each identifier in the user’s sample corresponds to one or many PEs in Reactome. An identifier corresponds to a PE if it matches with any of the identifiers stored in the different slots mentioned afore. In fact, the best way to solve this problem is by following the reverse approach; creating a lookup table with all the corresponding PEs per each identifier cross-referenced in Reactome. As a consequence, another important requirement is to minimise the memory usage so the data can be kept in memory to improve the query time.

The selection of a good data structure is then determined by requirements both to implement a fast lookup table and to keep memory usage low. A Trie is an ordered tree data structure that is used to store a dynamic set or associative array where the keys are usually strings [14]. A radix tree is a space-optimized Trie data structure where each node with only one child is merged with its parent [15].

**Fig. 1:**
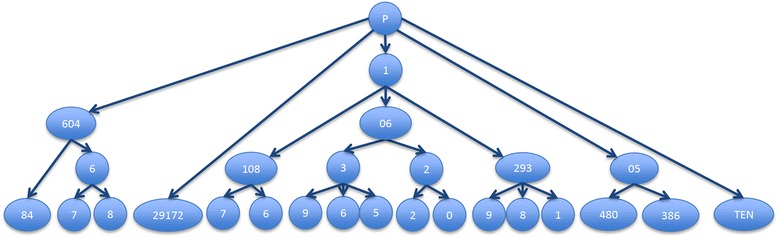
Radix tree representation for the identifiers P60484, P60467, P60468, P29172, P11087, P11086, P10639, P10636, P10635, P10622, P10620, P12939, P12938, P12931, P05480, P05386, PTEN

On the one hand, a radix tree has relatively low memory usage for the lookup table because the common prefixes are shared avoiding data duplication (Fig. 1). On the other hand, the cost of comparing a search key for equality with a key from the data structure can be a dominant cost which cannot be neglected. The radix tree string lookup algorithm fits the analysis algorithm’s original purpose because iterating over tree nodes keeps the identifier seeking time restricted to each identifier’s length and existence in the Reactome target set. As a consequence of this, in case the searched identifier is not contained in the data structure, there is no need to read all of it as happens in the hashing methods where the hash value of the string has to be calculated in every case by reading it entirely.

In summary, once a tree node is reached following the radix tree lookup algorithm for a given identifier, the presence or absence of references to PEs indicates whether the associated identifier is present or not in the database. Actually, the mentioned “references to PE” are indeed pointers to nodes in the data structure chosen for the next part of the analysis.

Reactome uses unique primary identifiers for the PEs it references, in particular UniProt for proteins and ChEBI for chemical entities. Thus, if users submit datasets using these reference systems, the mapping to PEs is straightforward. However, following frequent user requests, we also accept input data with non-unique identifiers, in particular gene names. These are then potentially mapped to multiple PEs. Thus, each target node in the tree could contain more than one pointer to the next data structure.

### Traversing complexes/sets composition and species projection

Reaching the associated single entity for a given identifier is the beginning of the second step in the analysis. When these single entities are part of a complex, they are also a target in this step of the analysis. Besides the single entities and complexes, there is another type of PE called sets which, along with complexes, are also to be considered. A set is an abstract representation of a group of two or more entities which are not interacting with each other but are functionally equivalent in the situation where the set is used, for example multiple members of a family of enzymes that could each potentially catalyse a reaction. Furthermore, complexes and sets can also contain other complexes and sets in order to represent much more elaborate structures causing the problem’s intricacy to grow.

Another specific requirement is the possibility of performing species projection to collect the results for *Homo sapiens* independently of the species for which the identifiers are provided, to benefit from the more complete Reactome annotation for Human. To do so, the species orthologs annotated in Reactome have to be taken into account. Orthologs are entities in different species that evolved from a common ancestor by speciation.

The last requirement in this step is to keep track of the identifiers mapping between the submitted identifiers and those used in Reactome to curate the single entities: UniProt accessions for proteins, Ensembl identifier for genes, CHEBI identifiers for small molecules and miRBase for microRNAs. Although an important part of this mapping started by including the known cross-references as identifiers in the radix tree in the previous step, the mapping itself has to be implemented in this step.

Summarising the exposed requirements for this step of the analysis, the chosen data structure has to model the entities composition problem, the species orthologs projection and the entities mapping. A directed graph is a graph, or set of nodes connected by edges, where the edges have a direction associated with them. For a given graph *G* with several nodes (*a*, *b*, *c* and *d*), if *G* has an arrow from *a* to *b* and another arrow from *b* to *c*, then the composed graph *G* ^*2*^ has an arrow from *a* to *c*. If *G* has an arrow from *a* to *b*, another arrow from *b* to *c* and yet another from *c* to *d*, then the composed graph *G* ^*3*^ has an arrow from *a* to *d*.

**Fig. 2:**
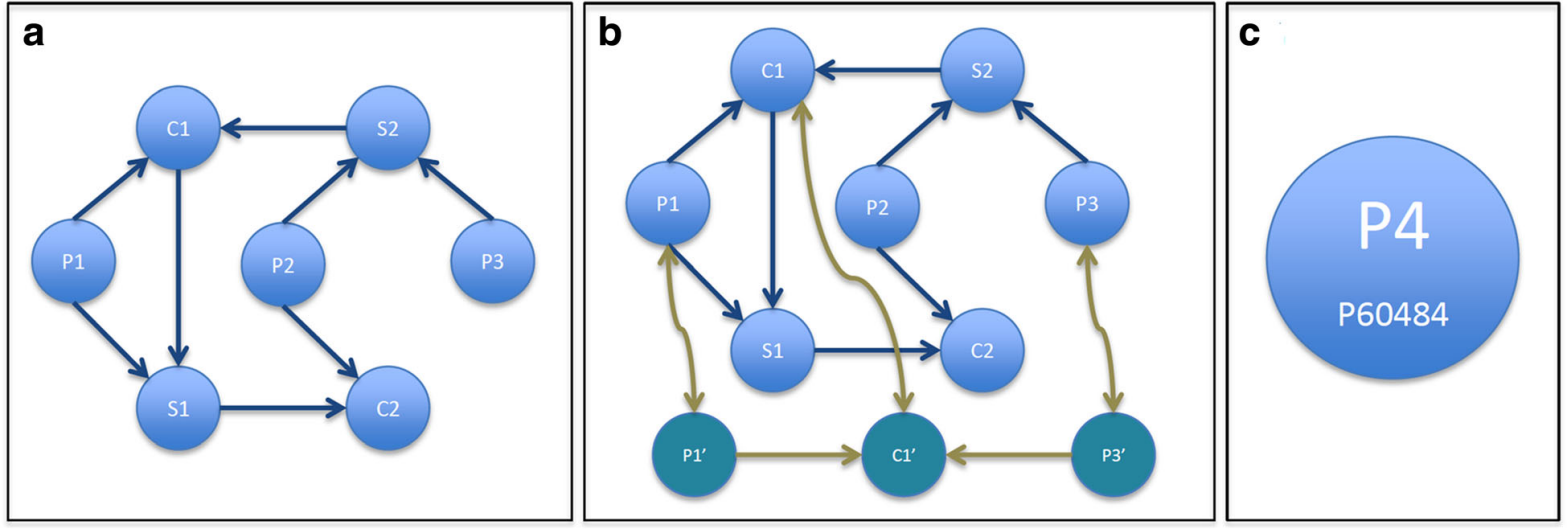
Graph representation where P are proteins; C are complexes, S are sets and prime nodes are the same but for other species. **a** One species graph. **b** Relation between two species. **c** Base node content

Building one graph per species (Fig. 2a) and interconnecting all of them linking all the ortholog nodes (Fig. 2b) creates a bigger graph where the projection requirement is then satisfied. Due to the node uniqueness in the final graph, for those cases where a node is part of one or more structured entities, it contains as many edges pointing to other graph nodes as structures in which it is contained, so structured entities are easily modelled. Finally, if each node of the graph contains its associated entity main identifier (Fig. 2c), when it is reached from a radix tree node representing an identifier other than the main one, this association is stored in order to be offered as part of the result as the required mapping once the analysis is finished.

The graph in Fig. 2a shows three proteins (P1, P2 and P3), two complexes (C1 and C2), and two sets (S1 and S2). By following the edge from node to node, S2 could be either P2 or P3, formally represented as [P2,P3]. C1 is a complex which, due to its edge from S2, is then potentially two complexes: {P1,P2} or {P1,P3}, represented as [{P1,P2},{P1,P3}]. Following this deconstruction, S1 is then [P1, {P1,P2}, {P1,P3}] and finally C2 is [{P1,P2}, {{P1,P2},P2}, {{P1,P3},P2}].

For instance, when an identifier matching with P3 is processed and its corresponding node in the graph is reached from the radix tree, it takes miniscule processing time to traverse the graph and reach the nodes S2, C1, S1 and C2. Likewise, if the target protein is P1, the reachable nodes following the graph edges are C1, S1 and C2. In both examples each target protein is part of the complexes and sets represented by the traversed nodes.

Employing a graph improves the analysis algorithm cost and, important in building an in-memory analysis, the memory usage is kept low because there is no data duplication as the node for a given main identifier is only in memory once. In addition, the final number of node iterations of the algorithm is limited by the related entities for a given identifier, avoiding queries against a large amount of data and intermediate results merging, as done in the database based approach.

As for the radix tree described above, the graph also requires a strategy to allow the algorithm to move on to the next analysis step. In this case, each graph node representing an entity directly associated to one or several pathways will contain as many links to the following data structure as different locations where it is present. Although in the current analysis step each entity associated with the target identifier is found, for the final result and the statistics calculation, there is still one more data structure to be used, as explained in the following sub-section.

### Results aggregation into the pathways organisation

Every PE that was directly or indirectly hit in the previous step is associated to one or more pathways. To calculate the significance of each pathway, for a given user sample, it is essential to determine the number of entities found per pathway. Due to the parent-child organisation of the Reactome pathways in an ontology-like hierarchy, when an entity is present in a certain pathway it is also present in its super-pathways in a recursive manner until a top-level pathway is reached (i.e. if a protein is present in “Metabolism of carbohydrates”, it is also present in “Metabolism”).

**Fig. 3:**
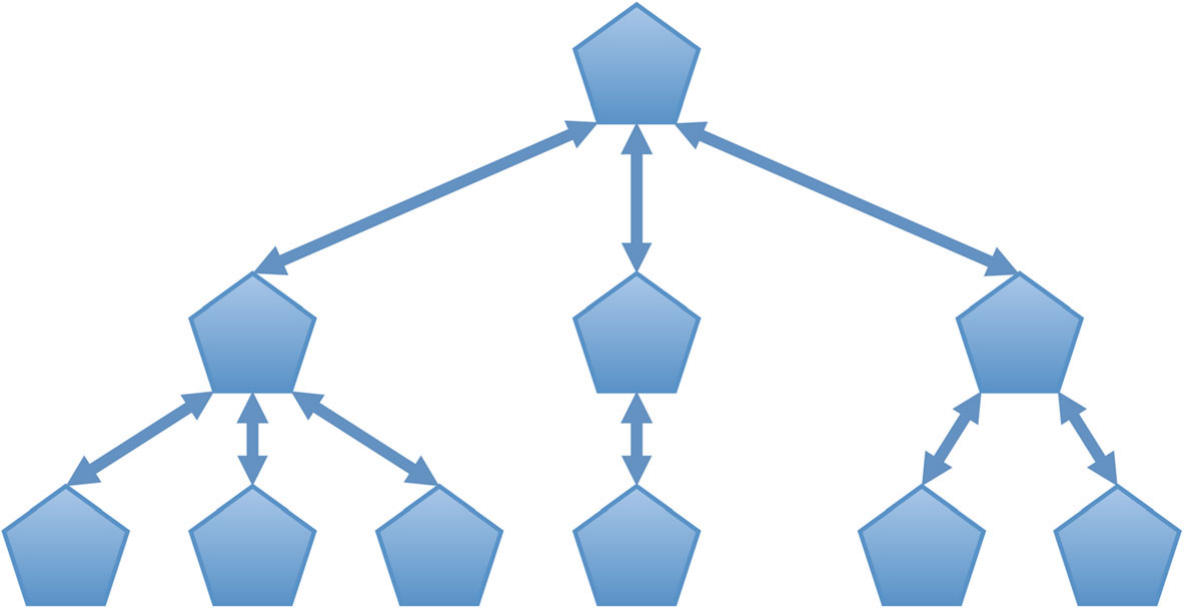
Double-linked tree to represent the event hierarchy in Reactome. The root node defines the species and its children represent the different pathways and sub-pathways in Reactome. Each node contains the pathway identifier, name, the total curated entities and the number of entities found in the user’s sample

Taking into account the requirements previously discussed, a good data structure to model this step is a double-linked tree, where each node represents a pathway and contains links to its parent and children (Fig. 3). When a node in the tree is hit, the action can be recursively propagated all the way up to the root. To reduce the memory footprint only identifiers, names and placeholders for results calculation are kept in each node.

Apart from being a convenient data structure to speed up collection of results and a good holder for the statistics results, once the analysis is finished, this data structure can also be serialised to a file to persist the result. In addition, associating the file to a token provides an easy way to create finer grained methods that allow filtering of the result on the server side to help speeding up light-weight clients. In this scenario, the clients can keep the token once the initial analysis is finished and depending on the user’s needs, perform several requests to the server referencing the associated token.

### Analysis result statistics calculation

The basic hypothesis in an over-representation analysis is that relevant pathways can be detected if the proportion of differentially expressed genes, within a given pathway, exceeds the proportion of genes that could be randomly expected [1]. Consequently, the fourth and last step in the analysis method involves the statistics calculation. This step does not require any extra data structure because the double-linked tree fits perfectly to the purpose.

The *p*-Value shows the statistical significance of each hit pathway for a given sample and the background for which the analysis has been performed. In Reactome the method used to calculate the statistical significance is the Binomial Test. Together with the *p*-Value, the False Discovery Rate (FDR) helps estimating the false positives and it is calculated using the Benjamini-Hochberg approach [16]. As mentioned afore, we have focussed on optimising the performance of the Reactome pathway analysis, while maintaining the basic algorithm as previously published [17].

## Results and discussion

This paper shows how splitting the pathway analysis method in four steps, in a way that every challenge can be easily addressed in a polynomial time using the appropriate data structures, speeds up the process and minimises the memory usage so the whole data structure can be kept in memory for a high-performance analysis. The result is a new set of analysis tools which vastly improve Reactome analysis interface performance and stability.

**Fig. 4:**
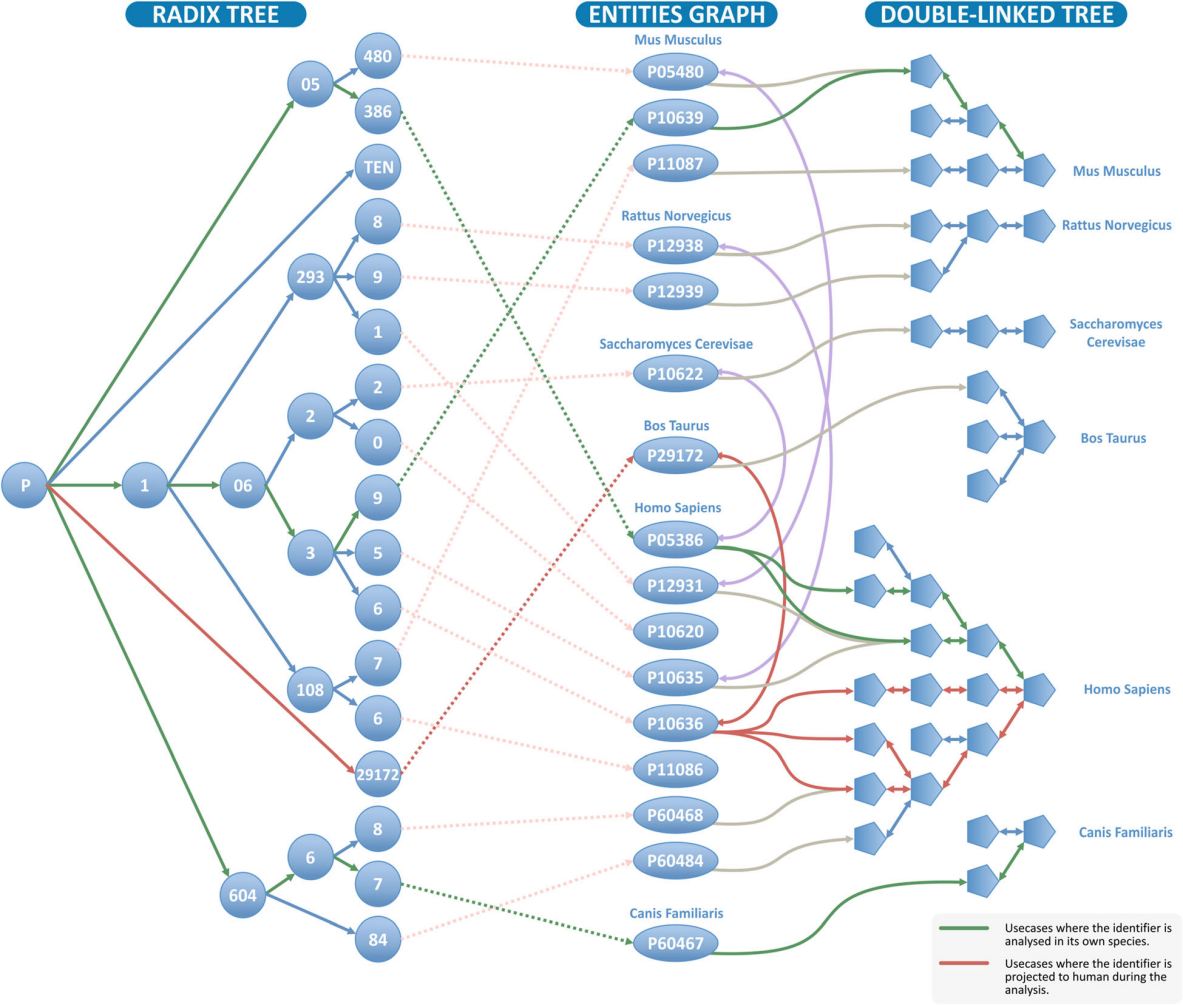
Representation of two analysis use cases joining the different data structures. In red an analysis performed using the projection to human. In green an analysis performed without projection

Summarising the steps (Fig. 4), for each identifier in the user’s sample, the first action is to find whether it is present in Reactome using a previously built radix-tree as a look-up-table. This speeds up the process, keeping a low memory footprint. For those that are present, the radix-tree nodes point to one or many nodes in a graph which is used as the second data structure to keep the curated relations between PEs as well as species orthology. Traversing this second data structure, applying or not the projection to species, provides pointers to all pathways stored in the final data structure, which is a double-linked tree, that helps aggregating the result and acts as a placeholder for the last step when the analysis statistics are calculated.

The described method has been developed using Java as programming language and can be downloaded from https://github.com/reactome/AnalysisTools. This package contains two main modules; *Core* and *Service*. The improved strategy has been developed in the *Core,* where the analysis is executed. The *Service* module is a Spring MVC (http://spring.io/) layer to create a RESTful service with a documented API, using OpenAPI, formerly known as Swagger v2.0 (http://swagger.io/), providing programmatic access. Hence, there are two ways of accessing the analysis tools; (1) programmatically via a web service (http://reactome.org/AnalysisService/) or (2) through a graphical user interface directly integrated in Reactome’s Pathway Browser (http://reactome.org/PathwayBrowser/#/TOOL=AT).

The web service is used to integrate the analysis in other system’s scripts, pipelines or to integrate the analysis in third-party applications. More information on how to do so can be found in Reactome’s developer zone (http://goo.gl/k5ffhu).

The pathway analysis approach described here is deployed in the Reactome production web site, stably handling on average 10.850 analysis requests from 2.000 unique users per month in the first half of 2016. Memory usage for the Apache Tomcat running this service plus other services in the server side is set to 2GB.

### Comparison with other resources

Among the plethora of pathway databases [1], there are resources with similar tools that perform over-representation analysis. Most notably, Gene Set Enrichment Analysis (GSEA) [18], the Database for Annotation, Visualization and Integrated Discovery (DAVID) [19], the Protein Analysis Through Evolutionary Relationships (PANTHER) [20] and ConsensusPathDB [21] are using similar statistical algorithms in their implementations and are freely available for academic use. Table 1 presents a comparison among these resources. For the comparison of processing time, only the first column in the four test sets, containing the gene identifiers, has been used. Reactome uses all genes annotated in the knowledge base as the background distribution. To our knowledge, this is also the approach used in the comparator tools, and we have not used options for custom background distributions, as statistics calculation could take longer in this scenario.

**Table 1:** Comparison of resources providing analysis methods and accessibility

Resource	Analysis methods	Online tool	Programmatic access	Processing time				
				Hippocampal atrophy - 79 genes^a^	Migraine disorder - 644 genes^b^	Parkinson’s disease - 1492 genes^c^	Multiple sclerosis - 2570 genes^d^	Inflammatory bowel disease - 4110 genes^e^
PANTHER	ORA	✔	–	~2 s	~4 s	~6 s	~8 s	~12 s
Consensus PathDB	ORA	✔	SOAP/WSDL	~1 min	~1 min	~3 min	~3 min	~1 min
DAVID	ORA	✔	SOAP/WSDL	~4 s	~4 s for conversion of official gene ids to 7498 DAVID ids. Analysis not performed - sample size limitation	~5 s for conversion of official gene ids to 17272 DAVID ids. Analysis not performed - sample size limitation	~8 s for conversion of official gene ids to 29420 DAVID ids. Analysis not performed - sample size limitation	Not performed - sample size limitation
GSEA	ORA	–	–		–	–	–	–
REACTOME v1.0	ORA	✔	–	~2 min	~7 min	~12 min	~19 min	~25 min
REACTOME v2.0	ORA	✔	REST	~1 s	~1 s	~2 s	~2 s	~3 s

Comparison between different resources and whether they provide analysis methods which are accessible online (UX or programmatic access) and the average response time for a predefined sample. For the comparison of processing time, only the first column in the test sets -the gene identifiers- has been used. Datasets are available in

^a^ https://www.targetvalidation.org/disease/EFO_0005039/associations (accessed 13/07/2016)

^b^ https://www.targetvalidation.org/disease/EFO_0003821/associations (accessed 13/07/2016)

^c^ https://www.targetvalidation.org/disease/EFO_0002508/associations (accessed 13/07/2016)

^d^ https://www.targetvalidation.org/disease/EFO_0003885/associations (accessed 13/07/2016)

^e^ https://www.targetvalidation.org/disease/EFO_0003767/associations (accessed 13/07/2016)

GSEA offers its analysis tool exclusively through a desktop application and therefore requires download and installation before usage, rendering the tool suitable for more experienced users. On the other hand, DAVID, PANTHER and ConsensusPathDB provide online access to their analysis tools via a web interface, similarly to REACTOME. Thus, users can submit their sample for analysis through their favourite web browser.

Furthermore, besides REACTOME, DAVID and ConsensusPathDB are also allowing users to access their analysis tools programmatically, through a set of web services. Hence, researchers and software developers can integrate the provided analysis tools into their pipelines and applications. However, while DAVID and ConsensusPathDB rely on the Simple Object Access Protocol (SOAP) and the Web Service Description Language (WSDL) for their web services, Reactome analysis web service is based on the Representational State Transfer (REST). The adoption of REST eliminates the need for complex clients and renders Reactome analysis service simpler, more lightweight, more flexible, and, thus, easier to integrate into third party software compared to its SOAP/WSDL counterparts.

Leveraging on the performance gained by the in-memory analysis approach explained above and the use of RESTful web services, the Reactome analysis tool does not impose any limitations on the sample size or the frequency of analysis requests, unlike DAVID. Regarding its weaknesses compared to DAVID, Reactome analysis tool has a more limited coverage, as it does not integrate as many resources as DAVID does, but it focuses on high quality manually curated pathways that are updated quarterly. In addition, Reactome does not allow users to customise the background population of their analysis.

## Conclusions

Through the use of highly optimised, in-memory data structures and algorithms, Reactome has achieved a stable, high performance pathway analysis service, enabling the analysis of genome-wide datasets within seconds, allowing interactive exploration and analysis of high throughput data.

## Availability and requirements

All data generated or analysed during this study are included in this published article.

Source code: https://github.com/reactome/AnalysisTools

Web service: http://reactome.org/AnalysisService/

User interface: http://reactome.org/PathwayBrowser/#/TOOL=AT

Documentation: http://goo.gl/k5ffhu

## Abbreviations

API: Application program interface
DNA: Deoxyribonucleic acid
FDR: False discovery rate
MVC: Model view controller
NP: Non-polynomial
ORA: Over-representation analysis
PE: Physical entities
REST: Representational State Transfer
RNA: Ribonucleic acid
SOAP: Simple object access protocol
SQL: Structured query language
WSDL: Web Service Description Language
